# Modeling Shrinkage and Creep for Concrete with Graphene Oxide Nanosheets

**DOI:** 10.3390/ma12193153

**Published:** 2019-09-26

**Authors:** Zengshun Chen, Yemeng Xu, Jianmin Hua, Xiao Zhou, Xu Wang, Lepeng Huang

**Affiliations:** 1 School of Civil Engineering, Chongqing University, Chongqing 400033, China; zengshunchen@cqu.edu.cn (Z.C.); xu.ym@cqu.edu.cn (Y.X.); hjm191@163.com (J.H.); zhouxiaocqjtu@gmail.com (X.Z.); 2 School of Civil Engineering, Chongqing Jiaotong University, Chongqing 400074, China

**Keywords:** shrinkage and creep, GO nanosheet, concrete, ACI209 model

## Abstract

In this study, the shrinkage and creep of concrete containing graphene oxide (GO) nanosheets were experimentally and theoretically investigated. Experiments for the shrinkage and creep of concrete with 0.02% and 0.08% GO nanosheets by the weight of cement and common concrete were carried out. Subsequently, the influence of GO nanosheets on the shrinkage and creep of concrete was analyzed and discussed. A modified model was developed to accurately predict the shrinkage and creep of concrete containing GO nanosheets after models for predicting shrinkage and creep of common concrete were compared and the influential factors and application scope were determined. Results indicate that: (1) GO nanosheets can increase the shrinkage strain and reduce the creep coefficient of concrete, and (2) a modified ACI209 (92) model can accurately predict the shrinkage and creep of concrete containing GO nanosheets. Factors considering concrete strength can be introduced in the model to improve the model accuracy.

## 1. Introduction

Concrete has been extensively applied to the building field, owing to its advantages such as easy material obtaining and strong plasticity, since it was invented [1,2,3]. However, common concrete continues to retain the complexities of early age behavior, such as low intensity, easily cracking, and so on [4]. In order to improve performance, silica fume [5], fiber material [6,7], and nanometer materials [7,8,9,10,11] are added to common concrete. Shah et al. [6] assessed the performance of a fiber reinforced concrete by using the ‘ring-test’, meanwhile the authors put forward an equation to estimate the residual stress in the concrete ring specimen. Hogancamp et al. [7] quantified the mechanical properties and cracking resistance of carbon nanofibers and carbon nanotubes in Portland cement pastes and mortars. Hawreen et al. [9] investigated the long-term shrinkage and creep effects, compressive strength, and modulus of elasticity of carbon nanotube reinforced concrete. Shaha et al. [10] reviewed the effects of nanomaterials on cement-based materials in recent years, and they found that nanomodification of cementitious materials could significantly improve the mechanical property, compactness, and durability. Panda et al. [11] explored the effects of nano attapulgite clay on static yield stress and viscosity recovery on high volume fly ash, which were useful for 3D concrete printing.

In addition, the 2D nanometer material graphene oxide (GO) nanosheet is one of the added materials that has received much attention [12]. As the derivative of graphene, GO is the carbon atom layer of ortho-hexagonal flake crystal structure including functional groups like hydroxyl radical, epoxy group, carboxyl, and carbonyl group, and it can be extended to dozens of micrometers along the lateral dimension [13]. Its structure approaches the plane and presents a 2-dimensional mesh. The surface of GO contains electronegative oxygen-containing groups, and the mutual repulsion between the same electric charges allows the GO to evenly spread in the water solution. Compared with graphene, it has better hydrophilia [14]. Its good humidity performance and surface activity can be stripped after intercalation of small molecules or polymers [15]. Hence, it plays a very important role in improving the comprehensive performance of materials like thermal property, electrical property, and mechanical property.

At present, there are many studies about the influence of GO nanosheets on the mechanical property and microstructure of cement and concrete [12]. Recent research has argued that the microstructure of cement composites is closely related to the compressive strength, bending strength, pore structure, and shrinkage value [16,17] and that GO nanosheets can improve the performance of cement composites. Tong et al. [18] investigated the use of GO nanosheets from three perspectives: A macro experiment, micro test, and atomic modeling. It was found that the GO nanosheet could improve the mortar strength, increase the corrosion resistance of binding materials, enhance the freezing and thawing performance of mortar, and make the cement paste form a more compact microstructure. Li et al. [19] found that the addition of 0.02%, 0.04%, 0.06%, and 0.08% GO nanosheet (by weight of cement) can increase the compressive strength of cement paste by 19.1%, 37.0%, 46.6%, and 46.8%, respectively, but the GO nanosheet with a dosage higher than 0.04% by weight of cement reduced the bending strength of cement paste. From the microcosmic perspective, they believed that one of the reasons might be that the GO nanosheet could make the cement paste more compact and reduce the porosity. Lv et al. [20] demonstrated that 0.03% GO nanosheet by weight of cement was the best addition to increase the strength of extension, compressive strength, and bending strength of cement composites. Yu et al. [5] found that GO nanosheets could reduce the mobility of cement paste and increase its yield stress and plastic strength. Chen et al. [21] observed the apparent structure through SEM and proved that the cracking and pores in the cement paste tended to reduce and shrink with the adding of GO nanosheets, making the microstructure more compact.

There are few studies about the influence of GO nanosheets on the shrinkage and creep performance of cement and concrete, and such research is still at an early stage. Xu et al. [12] believed that the adding of GO nanosheets would change the microstructure of cement composites and then influence the self-constriction, plastic shrinkage, drying shrinkage, and creep of the material. Pei et al. [22] found that with the increase of the GO nanosheet content, early-stage shrinkage strain of magnesium phosphate cement would drop first and then increase. The cement containing 0.06% GO nanosheet (by weight of cement) had the smallest shrinkage strain, and its peak strain dropped by 40.6% when compared with that of common magnesium phosphate cement.

Although there are many studies about the mechanical property [5,12,16,17,19,20], microstructure [12,16,17,21], and deformation performance [18,22] of concrete containing GO nanosheets now, the prediction model for the shrinkage and creep of concrete containing GO nanosheets is still not established, and there are only models for ordinary concrete. For instance, the model from the China Academy of Building Research (CABR) [23], the ACI209 series model [24,25], CEB-FIP series model [26], BS series model [27], GZ (1993) model [28], BP series model [29], and GL2000 model [30] have considered both the internal and external factors influencing concrete shrinkage and creep and predicted the shrinkage and creep of common concrete and light-weight aggregate concrete under specific conditions. However, all of the above models do not involve a prediction for the shrinkage and creep of concrete containing GO nanosheets. A small dosage of GO nanosheet can produce a huge influence on the cement and concrete [20,31,32]; it is therefore necessary to develop a model for predicting the shrinkage and creep of concrete containing GO nanosheets.

In order to investigate the shrinkage and creep effect of concrete containing GO nanosheets and to establish a shrinkage and creep model, the authors carried out experiments with concrete containing GO nanosheets.

## 2. Experimental Setups

### 2.1. Test Preparation

**Figure 1:**
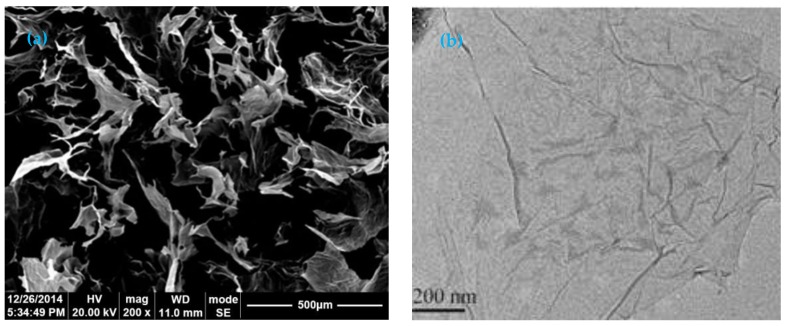
(**a**) Scanning electron microscope image of multilayer graphene oxide material; (**b**) transmission electron microscopy image of GO nanosheet.

The shrinkage and creep experiments of concrete containing GO nanosheets were performed in the Civil Engineering Material Lab of Chongqing University. The cement used for testing was ordinary Portland cement P.O 42.5, and the chemical components and percentage of the cement are shown in Table 1. The fine aggregate was machine-made sand with a fineness modulus of 1.64, and its specifications are shown in Table 2. Crushed limestone with a size range from 5 to 20 mm was used as the coarse aggregate. Furthermore, aggregate with a particle size of 5–10 mm accounted for 48%, while others accounted for 52%. The coal ash was Level II, and its chemical components are presented in Table 3. The chemical components of silica fume used in the experiment are shown in Table 4. Polycarboxylate superplasticizer was adopted as the water-reducing agent. The multilayer GO used in the experiment was provided by Suzhou Tanfeng Graphene Technology Co., Ltd. (Suzhou, China), and its appearance was black-brown powder. Composed of 5–10 layers of single GO sheet, it is characterized by a big diameter, small thickness, and large specific surface area. Its physical parameters and elemental properties are listed in Table 5. A scanning electron microscope (SEM) image of the GO nanosheet used in experiments is shown in Figure 1a. A 2D transmission electron microscope (TEM) image of the structure of GO nanosheet is shown in Figure 1b.

Figure 1a shows the SEM image of the multilayer graphene oxide material. It can be seen that the GO nanosheet structure almost completely fills the image. GO has a great specific surface area [14,15]. The surface area thereby provides a large interface between the cement matrix and the GO nanosheets when the GO nanosheets are mixed with the cement. In addition, GO exhibits a thin layered structure [14,15]. From the aspect of morphological characterization, it is a lamellar structure, which is a typical two-dimensional structural feature, and the thickness is thin due to the peeling of flake graphite. It can be seen from the surface of the sheet that the surface of the graphite after oxidation is still intact, and the area is on the micrometer scale and the thickness is on the nanometer scale.

Figure 1b is a TEM image of a multilayer GO sheet structure. The area shown in the figure is the overlapping structure of the multilayer GO nanosheets, and the wrinkled surface can form a mechanical interlock between the GO nanosheets and the cement matrix, which facilitates the load transfer between the interfaces to achieve higher bonding strength. GO nanosheet has a rough surface and there are many wrinkles which contain oxygen-containing functional groups [14]. Oxygen-containing functional groups greatly enhance the ability to absorb water molecules, which is why GO is more hydrophilic than graphene [5].

### 2.2. Sample Preparation and Test

The preparation method and sample size are determined according to the standard test methods of long-term performance and durability of ordinary concrete (GB/T 50082-2009). C50 concrete was prepared for the shrinkage and creep experiment, and the sample size was 100 mm × 100 mm × 400 mm. The water-cement ratio (w/c) was 0.35. According to previous studies [19,20,22], the addition of a low dose of GO has huge effects on the properties of concrete. Three groups of samples were prepared, and they were named PSC, GOSC-1, and GOSC-2, in which different amounts of GO nanosheets were incorporated as 0.02% and 0.08%, by weight of cement. Nine samples were prepared for each group. These samples were used for axial compression, shrinkage, and creep conditions, respectively, and each condition included 3 samples. The specific mixture ratio is presented in Table 6.

The material selection and their combinations were designed according to the standard test method of the mechanical properties of ordinary concrete (GB/T 50081-2002). In order to enable the graphene oxide powder to be dissolved and dispersed well in water, 0.02% and 0.08% GO-water solution was made first, and then the polycarboxylate superplasticizer was added to the graphene oxide aqueous solution. After that, the solution was added to concrete. The GO-water is tested using microscopic equipment used in a previous study [21]. Finally, the prepared solution was mixed with other cementitious material and aggregate and stirred. The slump of the concrete mixtures is around 20 mm. After mixing, the concrete mixture was loaded into the test mold. At the same time, the spatula was kept moving along the inner wall of the test mold to be sure that the mixture was raised above the test port. After the filling was completed, the test mold was placed on a vibrating table to vibrate until the surface was discharged. After the test piece was formed, the surface was covered with a water-impermeable film to prevent water from being dispersed. The test pieces were numbered and removed 24 h later and immediately placed in a standard curing room with a temperature of 20 ± 2 °C and a relative humidity of 95% or more.

**Figure 2:**
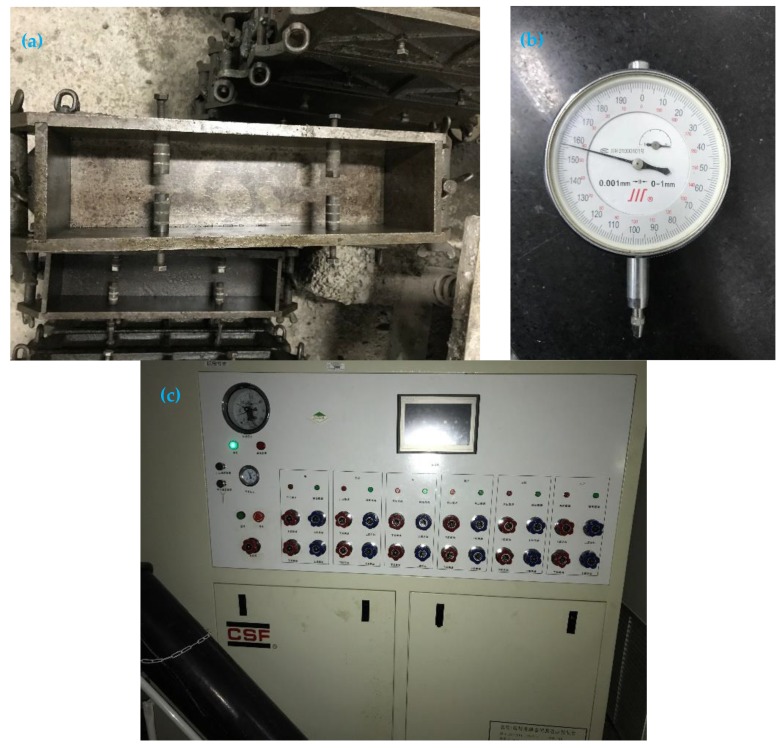
Laboratory apparatus: (**a**) embedded probe; (**b**) micrometer gauge; (**c**) static servo hydraulic creep loading device.

A deformation meter, as shown in Figure 2b, and a hydraulic creep loading device of automatic control, as shown in Figure 2c, were used for the creep experiment.

**Figure 3:**
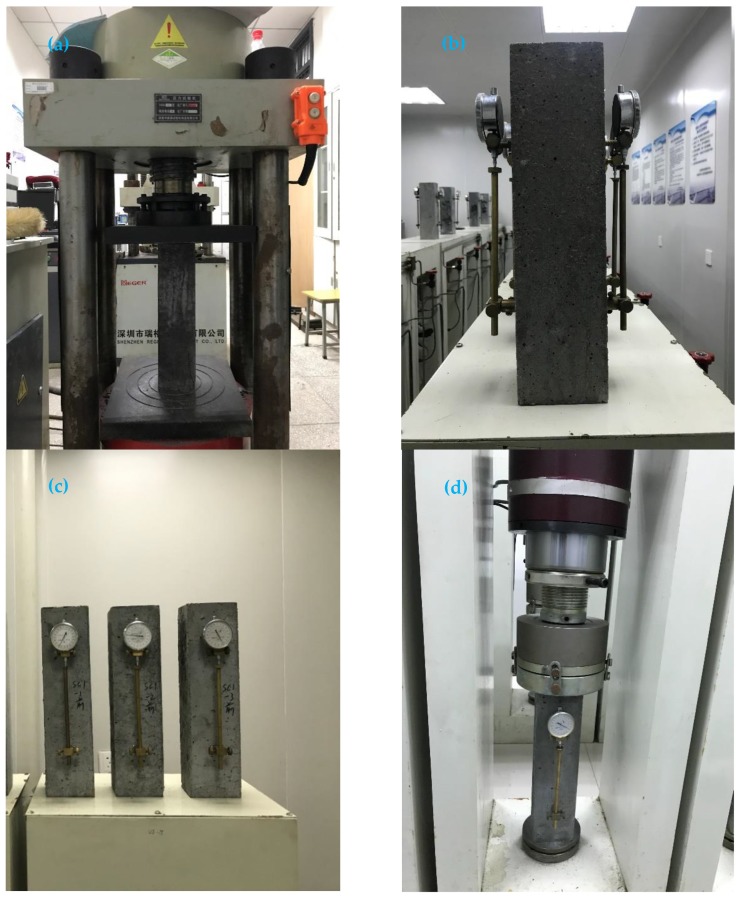
Experiment procedure: (**a**) concrete axial compression test; (**b**) micrometer gauge installation; (**c**) concrete shrinkage test; (**d**) concrete creep test.

The samples were maintained in the standard curing room for 7 days, and the axial compressive strength test was conducted for various components before the shrinkage and creep test, as shown in Figure 3a. The axial compressive strengths of PSC, GOSC-1, and GOSC-2 during the 7 days were 36.9 MPa, 38.0 MPa, and 42.0 MPa, and 40% of the axial compressive strength was considered as the creep stress. Furthermore, the axial compressive strengths during the 28 days were 56.9 MPa, 59.2 MPa, and 64.1 MPa.

After the axial compressive strength test was completed, the micrometer gauge was installed on the sides of the shrinkage and creep samples, as shown in Figure 3b. The initial reading of the shrinkage sample was recorded, as shown in Figure 3c. Centering was conducted for the creep samples under the creep stress of 20%. When the deformation values on both sides exceeded 10% of the average value, centering was conducted again after unloading. The load was increased to the creep stress immediately after centering, and the initial deformation values on both sides recorded, as shown in Figure 3d. Later, the deformation values of shrinkage and creep samples were recorded on the 1st, 3rd, 7th, 14th, 28th, 45th, 60th, 120th, and 150th days during the loading age.

## 3. Experimental Results

### 3.1. Influence of GO Nanosheet on Shrinkage of Concrete

Concrete shrinkage includes plastic shrinkage, autogenous shrinkage, drying shrinkage, and carbonated shrinkage. The shrinkage strain of the test samples was measured after 7 days of maintenance, so plastic shrinkage produced in the concrete molding and hardening process was not considered. Carbonated shrinkage often takes a long time, so it was also ignored. The entire shrinkage process in the experiment included autogenous shrinkage and drying shrinkage. Autogenous shrinkage is the macro volume shrinkage triggered by self-desiccation in the concrete when the concrete has no moisture exchange with the outside environment. Drying shrinkage refers to shrinkage caused by water loss in the environment.

The shrinkage value gained in the experiment was processed according to the following equation, and the concrete shrinkage was gained:

(1) $$\varepsilon_{st}\;=\;\frac{L_{0}\;-\;L_{t}}{L_{b}}$$

where *ε_st_* is the shrinkage rate when the test period of concrete sample is *t(d)*, and t is calculated from the initial start of the test; *L_b_* is the scale distance of concrete sample (400 mm in this experiment); *L_0_* is the initial reading of sample length (mm); *L_t_* is the length reading of concrete sample when the test period is *t*(*d*) (mm).

**Figure 4:**
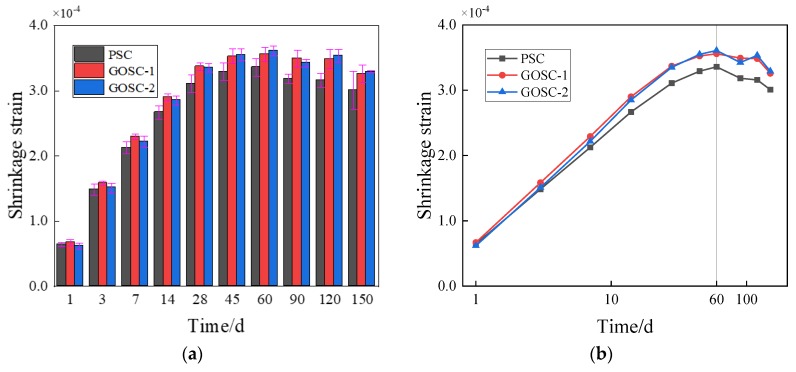
Changes of the shrinkage strain of concrete with time. (**a**) Variability of shrinkage; (**b**) trend graphic in which the age of the concrete uses a logarithmic scale.

Figure 4a presented the variability of shrinkage. Among all the standard deviations, the value of PSC on the 150th day reached the peak value, which was 0.000029, and the coefficient of variation was 0.096, which was also the peak value.

The change of the shrinkage strain of concrete with time was obtained from the test, as shown in Figure 4b. It shows that the shrinkage strain of all concrete samples increases with the increase of loading age in the first 60 days. According to the experimental data, the shrinkage strains of PSC, GOSC-1, and GOSC-2 on the first day are similar (0.000064, 0.000067, and 0.000062), while the GO nanosheets of various dosages have different influences on the shrinkage strain of concrete. On the one hand, GO nanosheets can hinder the development of microcracks [33]. On the other hand, GO nanosheets can fill in the microcracks which prevent free water flowing to an external dry environment [34]. The shrinkage strain becomes larger as the amount of residual free water increases [35]. When the dosage of GO nanosheets is relatively high (0.08% by the weight of cement), the first reaction happens, which reduces the shrinkage strain. When the dosage of GO nanosheets is relatively low (0.02% by the weight of cement), the second reaction happens, which increases the shrinkage strain.

The shrinkage strain of concrete with GO nanosheets was higher than the shrinkage strain of concrete without GO nanosheets after the 3rd day. This is possibly because when GO nanosheets were mixed with cement in the beginning, the electronegative oxygen functional groups in GO nanosheets and the metal cations in the cement compound adsorbed each other rapidly, and the formation of flocculent structure reduced the mobility of the cement [5]. Such nucleation will accelerate cement hydration [8]. Moreover, in the early-stage hydration process, CaO in the cement paste is hydrated to generate Ca(OH)_2_, which will produce a chemical reaction with GO(–COOH) in graphene. Hence, the hydration process is accelerated [36,37], the total absolute volume of the cement system is reduced, and the shrinkage strain rises.

On the 60th day, the shrinkage strain of the three groups reached the peak value at the same time, and the values increased with the increase of the addition of the amount of GO nanosheets. After the 60th day, the shrinkage strain began to slowly decline. In the later hydration process, the increase rate of shrinkage strain dropped gradually. This is because: (1) the moisture adsorbed by GO nanosheets is released, which will help to solidify the concrete [38] and to weaken autogenous shrinkage; (2) a chemical bond might be formed between GO nanosheets and cement [39], which will reduce the drying shrinkage rate; (3) GO nanosheets will promote the concrete to form a compact microstructure [18]; hence, the cement paste rigidity will increase, and its shrinkage will be restricted.

### 3.2. Influence of GO Nanosheets on Creep of Concrete

Equation (2) was used to calculate the creep strain of concrete.

(2) $$\varepsilon_{ct}\;=\;\frac{L_{t}\;-\;L_{0}}{L_{b}}\;-\;\varepsilon_{t}$$

where *ε_ct_* is the creep strain (mm/m) when the concrete samples are loaded for t days, and it is accurate to 0.001 mm/m; *L_t_* is the total deformation value (mm) when the concrete samples are loaded for t days, and it is accurate to 0.001 mm; *L*_0_ is the initial deformation value (mm) in loading, and it is accurate to 0.001 mm; *L_b_* is the scale distance (mm), and it is accurate to 1 mm; *ε_t_* is the shrinkage value (mm/m) of the shrinkage samples under the same age, and it is accurate to 0.001 mm/m.

The creep coefficient was used to measure the creep behavior of concrete with GO nanosheets, and is expressed by:

(3) $$\varphi_{t}\;=\;\frac{\varepsilon_{ct}}{\varepsilon_{0}}$$

(4) $$\varepsilon_{0}\;=\;\frac{L_{0}}{L_{b}}$$

where *φ_t_* is the creep coefficient after loading for *t* days, and it is accurate to 0.001 mm/m; *ε*_0_ is the initial strain value (mm/m) in loading, and it is accurate to 0.001 mm/m.

**Figure 5:**
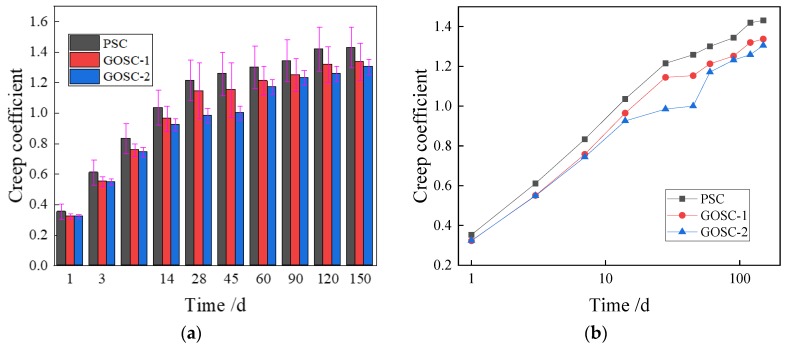
Changes of concrete creep coefficient with time. (**a**) Variability of creep coefficient; (**b**) trend graphic in which the age of the concrete uses a logarithmic scale.

Figure 5a presents the variability of the creep coefficient. The standard deviation of GOSC-1 reached the peak value on the 28th day, which is 0.183. Moreover, the peak value of the coefficient of variation was 0.140, which appeared on the 1st day of PSC.

In Figure 5b, the creep coefficient of the three groups increased with time. Furthermore, the change of the creep coefficient of concrete with different dosages of GO nanosheets with time is similar. The creep coefficient increases fast in the first 14 days, indicating that the change rate of strain is relatively high. However, the curve is comparatively gentle after 14 days, and the slope also drops obviously, showing that the change rate of the creep coefficient is relatively low.

**Figure 6:**
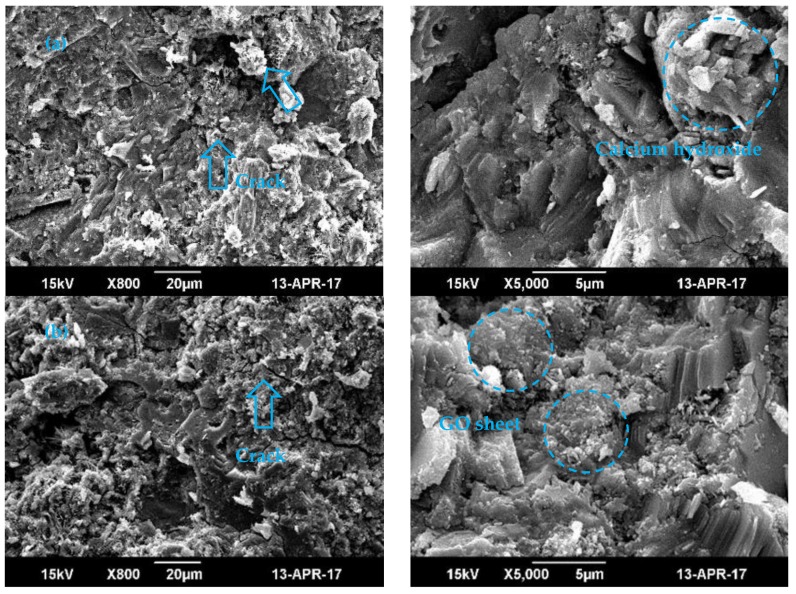
SEM image of the test piece [22]: (**a**) ordinary cement; (**b**) GO cement (0.06%).

According to the comparison of the creep coefficient curve of different concrete samples, the creep coefficient of the GOSC-1 and GOSC-2 groups are obviously lower than that of the PSC group, indicating that the addition of GO nanosheets can significantly reduce the creep coefficient of concrete. Moreover, the creep coefficient of samples with a larger dosage is smaller than the creep coefficient of samples with a smaller dosage, as GO nanosheets can enhance the compressive strength of concrete. Furthermore, within a certain scope, the higher the GO nanosheets content is, the bigger the compressive strength will be [20,40] and the smaller the creep coefficient will be. In addition, the reduction rate of the creep coefficient of concrete with 0.08% GO nanosheets by the weight of cement is higher than that of concrete with 0.02% GO nanosheets by the weight of cement. The creep coefficient of concrete with 0.08% GO nanosheets by the weight of cement drops the most obviously on the 28th and 45th days, and the reduction rates of the creep coefficient are 18.93% and 20.53% respectively. On the 60th day, the reduction rates of the creep coefficients of concrete with 0.02% and 0.08% GO nanosheets by the weight of cement are 6.73% and 9.93%. The possible reason is that GO nanosheets can form a compact microstructure through template effect and self-assembly effect in the cement hydration process [41] (see Figure 6) and transfer the crack energy into the cement matrix to restrain the extension of cracks [38]. Therefore, the higher the dosage of GO nanosheets is, the smaller the creep strain of concrete will be.

A previous study [22] can also explain the experimental results. According to the SEM image presented in Figure 6, cracks and pores are obviously distributed in common cement paste. With the addition of 0.06% GO nanosheets by the weight of cement, cracks and pores in the cement paste tend to decrease, and a compact microstructure is formed. Therefore, the higher the dosage of GO nanosheet is, the smaller the creep strain of the concrete will be. Furthermore, the increase in the rate of the shrinkage strain of GO nanosheet concrete increases with the increase of age in the early stage.

## 4. Modeling Shrinkage and Creep for Concrete Containing GO Nanosheets

### 4.1. Traditional Models for Predicting Shrinkage and Creep of Concrete

#### 4.1.1. Comparison of Factors Considered in Common Models

Traditional models for predicting shrinkage and creep of common concrete are presented in Appendix A. They have been developed according to a lot of test data. Because of the limitation of test conditions and different research emphases, various models have considered different factors. Table 7 shows the influential factors of existing models.

As can be seen from Table 7, the above models consider external factors more to predict shrinkage and creep of common concrete, especially the CABR model (1986), which does not consider internal factors at all. Moreover, all the models consider loading age, calculating age, applied stress, concrete 28d compressive strength, component section size, and relative humidity. Among all the internal factors, cement type is considered the most important factor, as both the CEB-FIP model (1978 and 1990) and GL2000 model (2000) take it into account.

#### 4.1.2. Application Scope of Traditional Models

As mentioned previously, traditional models were established based on test data that were obtained in certain test conditions. Accordingly, these models have their own application scope. Details of the application scope are summarized in Table 8.

### 4.2. Comparison of Shrinkage and Creep between Model Calculation and Experimental Result

**Figure 7:**
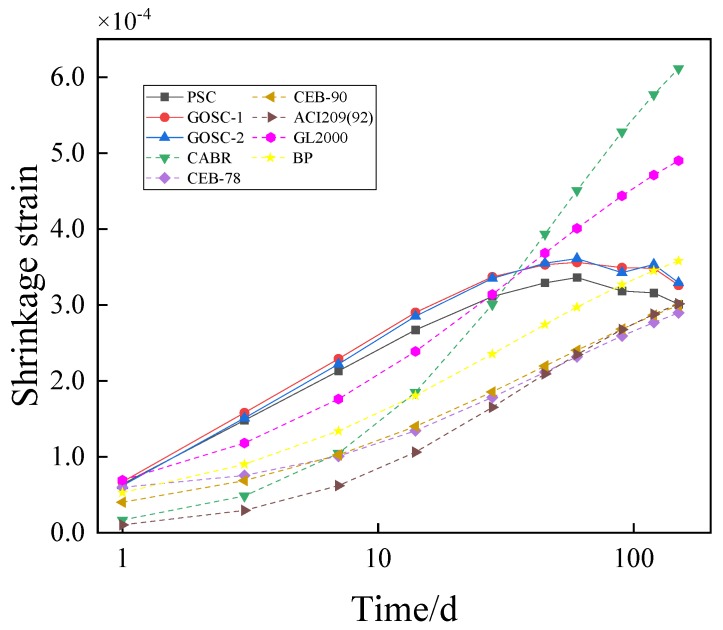
Comparison of the shrinkage strain between calculations and experimental results of the C50 concrete. (Note: The age of the concrete uses a logarithmic scale.).

**Figure 8:**
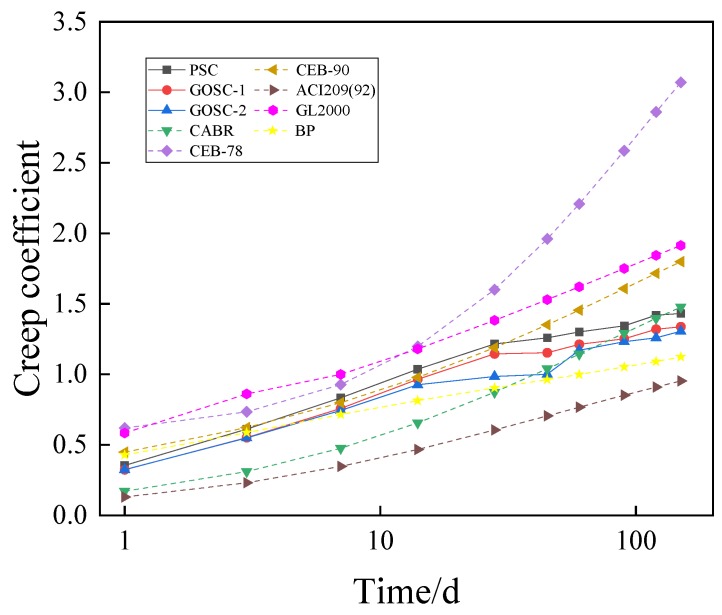
Comparison of the creep coefficient between calculations and experimental results of the C50 concrete. (Note: The age of the concrete uses a logarithmic scale).

The test conditions documented in Section 3 were substituted into the traditional models (Appendix A) to obtain the shrinkage and creep of the concrete containing GO nanosheets. The values were compared with the experimental results, as presented in Figure 7 and Figure 8.

In Figure 7, the theoretical calculated values of CEB-78, CEB-90, and BP are smaller than the experimental values. The differences between the theoretical calculated values of the China Academy of Building Research, ACI209(92) and GL2000, and the measured values present different rules in different periods. Before the 28th day, the theoretical calculated values are smaller than the measured values. However, after the 28th day, the theoretical calculated values begin to exceed the measured values. As for the reason, concrete with coal ash and silica fume shows greater initial shrinkage than common concrete, but the existing models seldom consider the adding of coal ash and silica fume. As a result, the error between the theoretical calculated values and measured values is relatively big. In Figure 8, the theoretical calculated values of CEB-78 and GL2000 are greater than the measured values, while the theoretical calculated values of China Academy of Building Research, BP and ACI209 (92) are smaller than the measured values. The theoretical calculated value of CEB-90 fits the measured value well before the 28th day, while it is greater than the measured value after the 28th day.

### 4.3. Model Modification and Analysis

Figure 7 and Figure 8 show that the trends of the values predicted by ACI209 model are in close agreement with those of the experimental values. Moreover, the ACI 209 model established by the American Concrete Institute (ACI) has the following advantages: (1) its parameters are formulized; (2) computer calculation is relatively easy; (3) numerous factors of concrete shrinkage and creep are considered; and (4) the loading time is presented with a hyperbolic curve. In addition, the influential factors of the concrete shrinkage and creep can be divided into internal factors and external factors. Internal factors include cement variety, water cement ratio, mineral admixture and aggregate, additive, etc.; external factors include loading time, loading stress, temperature, humidity, etc. According to the shrinkage and creep of concrete containing GO nanosheets, the final values of shrinkage strain and creep coefficient with different strengths are different. Therefore, the author considered the influence of concrete strength based on the ACI209 model, and introduced the influence coefficients of concrete strength on the final values of shrinkage and creep (i.e., $\gamma_{\mathrm{st}}^{\mathrm{sh}}$ and $\gamma_{\mathrm{st}}^{c}$) as well as the exponential influence coefficient of concrete strength on the shrinkage and creep development curves (i.e., *α* and *β*). Accordingly, models for predicting shrinkage and creep of concrete containing GO nanosheets are modified by:

(5) $$\varepsilon_{sh\left(t\right)}\;=\;\gamma_{st}^{sh}\varepsilon_{sh\infty }{\left(\frac{t}{35\;+\;t}\right)}^{\alpha }$$

(6) $$\varphi_{\left(t,\tau \right)}\;=\;\gamma_{sh}^{c}\varphi_{\infty }{\left[\frac{{\left(t\;-\;\tau \right)}^{0.6}}{10+{\left(t\;-\;\tau \right)}^{0.6}}\right]}^{\beta }$$

where *ε_sh∞_* and *φ_∞_* are the final values of shrinkage strain and creep coefficient in the ACI209 model.

The 1st Opt fitting software was used to fit the concrete containing 0.02% and 0.08% GO nanosheets by weight of cement to get the coefficients in Equations (5) and (6). The influential and exponential coefficients obtained from the weighting are presented in Table 9 and Table 10.

**Figure 9:**
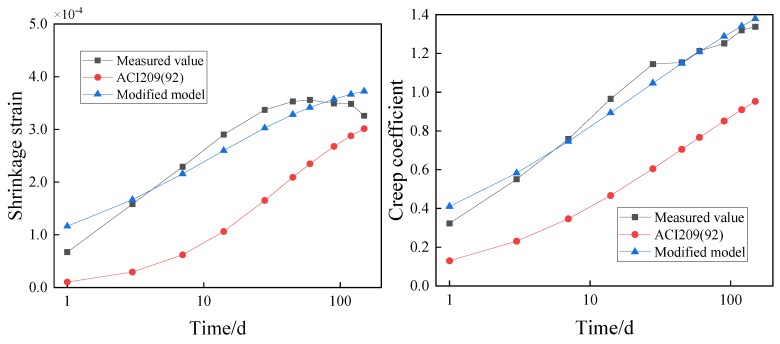
Comparison of shrinkage and creep models of concrete containing 0.02% GO nanosheets by weight of cement. (Note: The age of the concrete uses a logarithmic scale).

**Figure 10:**
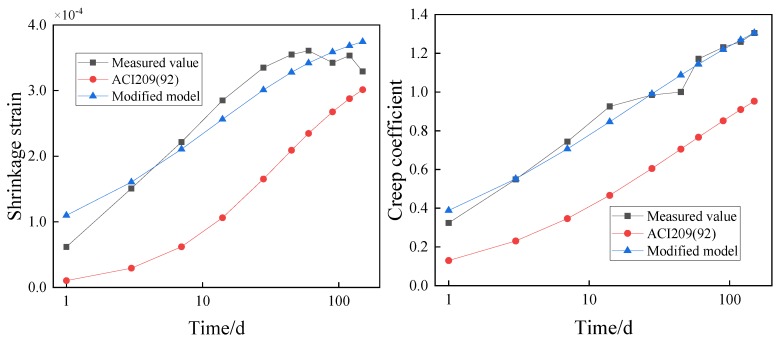
Comparison of shrinkage and creep models of concrete containing 0.08% GO nanosheets by weight of cement. (Note: The age of the concrete uses a logarithmic scale.).

Using the modified model, the shrinkage and creep of the concrete containing 0.02% and 0.08% GO nanosheets by weight of cement are calculated and compared with that predicted by the ACI 209 (92) model and the experimental result, as shown in Figure 9 and Figure 10.

In Figure 9 and Figure 10, the values of the shrinkage and creep predicted by the modified model are in close agreement with the experimental results, while there are large discrepancies between the values predicted by the ACI 209 (92) model. The results suggest that the modified model can be used for predicting shrinkage and creep of concrete containing GO nanosheets under the same test conditions. 

For the purpose of convenient use, the coefficients in Equations (5) and (6) are modeled. Because of the different fundamental characteristics of concrete, such as cement variety, water cement ratio, mineral admixtures, and aggregates, the concrete shrinkage and creep are different. Meanwhile, the differences of test conditions will also lead to the differences of concrete shrinkage and creep. Therefore, the coefficients of concrete strength influence the final values of shrinkage and creep as well as the relation between the concrete strength at 28 days and shrinkage and creep development curve indexes. Linear square fitting was conducted according to the values in Table 9 and Table 10, and the fitting results are obtained by:

(7) $$\gamma_{st}^{sh}\;=\;1.044973\;+\;0.002316f_{cm};\;\gamma_{st}^{c}\;=\;2.628846\;-\;0.017414f_{cm}$$

(8) $$\alpha \;=\;0.123510\;+\;0.003749f_{cm};\;\beta \;=\;0.608063\;-\;0.000008f_{cm}$$

where *f_cm_* is the average cube crushing strength of concrete in 28 days.

The above modeled coefficients were introduced into Equations (5) and (6) to obtain Equations (9) and (10), which can be used for modeling the shrinkage and creep of concrete containing GO nanosheets.

(9) $$\varepsilon_{sh\left(t\right)}\;=\;\left(1.044973\;+\;0.002316f_{cm}\right)\varepsilon_{sh\infty }{\left(\frac{t}{35\;+\;t}\right)}^{\left(0.12310\;+\;0.003749f_{cm}\right)}$$

(10) $$\varphi_{\left(t,\tau \right)}\;=\;\left(2.628846\;-\;0.017414f_{cm}\right)\varphi_{\infty }{\left[\frac{{\left(t\;-\;\tau \right)}^{0.6}}{10\;+\;{\left(t\;-\;\tau \right)}^{0.6}}\right]}^{\left(0.608063\;-\;0.000008f_{cm}\right)}$$

## 5. Conclusions

This study has compared the shrinkage and creep of concrete containing 0.02% and 0.08% GO nanosheets by weight of cement and common concrete. The shrinkage and creep on the 1st, 3rd, 7th, 14th, 28th, 45th, 60th, 120th, and 150th days during the loading age were experimentally obtained. This study has provided experimental evidence for other related investigation. Furthermore, the existing models for the shrinkage and creep of common concrete have been modified to predict the shrinkage and creep of concrete containing GO nanosheets, which will provide more reference for other researchers. The main conclusions are drawn as follows.

(1) GO nanosheets can increase the shrinkage strain and reduce the creep coefficient of concrete. The higher the dosage is, the larger the influence will be.

(2) The influence of GO nanosheets on the shrinkage strain of concrete increases first and then drops as the loading age rises. In a certain scope, with the increase of the dosage, it often takes a longer time to get a peak value.

(3) The influence of GO nanosheets on the creep of concrete does not present a unified changing trend in the loading age. However, in a certain scope, the higher the dosage of GO nanosheets is, the larger the influence on the creep coefficient will be.

(4) A modified ACI209 (92) model can accurately predict the shrinkage and creep of concrete containing GO nanosheets. Factors considering concrete strength can be introduced in the model to improve the model accuracy.

## Figures and Tables

**Table 1:** Chemical components and percentage of cement.

Chemical Component	SiO_2_	Fe_2_O_3_	Al_2_O_3_	CaO	MgO	K_2_O	Na_2_O	SO_3_	Loss on Ignition
**Percentage (%)**	21.97	3.36	6.36	61.22	1.82	0.98	0.22	3.00	4.48

**Table 2:** Grain composition of fine aggregate.

Nominal Diameter (mm)	0	0.15	0.3	0.6	1.18	2.36	4.75
**Accumulative Sieve Residue (%)**	100	89.54	75.1	53.16	38.48	19.44	1.06

**Table 3:** Chemical components and percentage of fine aggregate.

Chemical Component	SiO_2_	Fe_2_O_3_	Al_2_O_3_	CaO	MgO	SO3	K_2_O	Na_2_O	Loss on Ignition
**Percentage (%)**	45.50	12.96	26.23	2.39	2.35	1.72	1.66	0.94	3.00

**Table 4:** Chemical components and percentage of silica fume.

Chemical Component	SiO_2_	Fe_2_O_3_	Al_2_O_3_	MgO	Na_2_O	Loss on Ignition
**Percentage (%)**	87.96	0.7	0.85	1.41	0.52	4.9

**Table 5:** Physical and chemical properties of GO nanosheets (kg/m^3^).

Appearance	Physical Parameter					Chemical Component (XRD Test)		
	Purity	Thickness	Slice Layer Diameter	Number of Layers	Specific Surface Area	C	O	S
Black-brown Powder	>95 wt.%	3.4–8 nm	10–50 μm	5–10	100–300 m^2^/g	68.44%	30.92%	0.63%

**Table 6:** Experiment number and mixture ratio (kg/m^3^).

Number	Cement	Coal Ash	Silica Fume	Machine-made Sand	Coarse Aggregate	GO	Water	Polycarboxylate Superplasticizer
PSC	370	70	28	723	1085	0	164	6.08
GOSC-1	370	70	28	723	1085	0.074	164	6.08
GOSC-2	370	70	28	723	1085	0.296	164	6.08

**Table 7:** Comparison of factors considered in common shrinkage and creep prediction models.

Factors	Model Factors	CABR (1986)	ACI209 (1992)	CEB-FIP (1978)	CEB-FIP (1990)	BP (1978)	GL2000 (2000)
Internal Factors	Weight Ratio of Aggregate to Cement						
	Air Content		√				
	Cement Content					√	
	Cement Type			√	√		√
	Concrete Concentration		√				
	Fine Aggregate Accounts for Aggregate Weight		√				
	Slump		√				
	Water Cement Ratio						
	Water Content						
External factors	Loading Age	√	√	√	√	√	√
	Calculating Age	√	√	√	√	√	√
	Applied Stress	√	√	√	√	√	√
	Concrete Characteristic Strength during Loading					√	
	Cross Section Strength						
	Maintenance Method	√					
	Concrete 28d Compressive Strength	√	√	√	√	√	√
	Load Duration		√	√	√	√	√
	Component Section Size	√	√	√	√	√	√
	Concrete Elastic Modulus during Loading						√
	Concrete 28d Modulus of Elasticity		√	√	√		
	Relative Humidity	√	√	√	√	√	√
	Environment Humidity						
	Component Dry Age					√	√

**Table 8:** Application scope of traditional model for predicting shrinkage and creep of concrete.

Prediction Model	Year	Application Scope
Model of China Academy of Building Research	1986	The pressure stress shall not exceed 0.5 *f_ck_* or 0.4 *f_cu,k_*, the strength grade of common concrete shall not exceed C40, the strength grade of light-weight aggregate concrete shall not exceed LC30, the relative humidity shall be 40–80%, and the environment temperature shall be about 20 °C. For common concrete, the applicable cement content shall be 225–413 kg/m^3^, the water cement ratio shall be 0.44–0.75, and the sand rate shall be 0.30–0.40. For light-weight aggregate concrete, the applicable cement content shall be 225–450 kg/m^3^, the water cement ratio shall be 0.40–0.76, and the sand rate shall be 0.40–0.55.
ACI209R (1992)	1992	The relative humidity shall be 40–80%; the cement of Type I and II shall be maintained in moisture for no less than 7 days and maintained in steam for no less than 1 day.
CEB-FIP (1978)	1978	When the component hardens under the constant environment, the stress shall not exceed 0.4 *f_cu,k_*. Concrete maintained under extremely high or low temperature and heat is not applicable.
CEB-FIP (1990)	1990	For common structural concrete, the compressive stress in the concrete age of loading shall be less than 0.4 *f_cu,k_*, the average compressive strength of concrete in 28 days shall be 20–90 MPa, the relative humidity shall be 40–100%, the average temperature shall be 5–30 °C, the cement of Type I, II, and III shall be maintained in moisture for less than 14 days and maintained in steam for less than 14 days, and tension concrete is allowed.
BP	1995	The average compressive strength of concrete in 28 days shall be 17.2–69 MPa, the aggregate/cement ratio shall be 2.5–13.5, the cement content shall be 160–719 kg/m^3^, the water cement ratio shall be 0.35–0.85, the relative humidity shall be 40–100%, and the drying situation before loading of cement of Type I, II, and III shall be calculated.
GL2000 Model	2000	The average compressive strength of concrete in 28 days shall be 16–82 MPa, the water cement ratio shall be 0.40–0.60, the relative humidity shall be 20~100%, and the drying situation before loading of cement of Type I, II, and III shall be calculated.

**Table 9:** Influential and exponential coefficients (concrete containing 0.02% GO nanosheets by weight of cement).

Coefficients	GO (0.02%)		
$\gamma_{st}^{sh}$	1.1821	$\gamma_{\mathrm{st}}^{c}$	1.59792
*α*	0.34545	*β*	0.60758
Residual Sum of Squares	8.15443 × 10^−9^	Residual Sum of Squares	0.02714

**Table 10:** Influential and exponential coefficients (concrete containing 0.08% GO nanosheets by weight of cement).

Coefficients	GO (0.08%)		
$\gamma_{st}^{sh}$	1.19345	$\gamma_{\mathrm{st}}^{c}$	1.51259
*α*	0.36382	*β*	0.60754
Residual Sum of Squares	8.1468 × 10^−9^	Residual Sum of Squares	0.02063

## Appendix A. Models for Predicting Shrinkage and Creep of Common Concrete

(1) Model of the China Academy of Building Research (1986)

The model first calculates the basic formula of shrinkage of ordinary concrete and lightweight aggregate concrete, and then comprehensively considers the various mechanical indexes of concrete and includes various influence factors of concrete shrinkage and creep. Although the model considers many factors, the applicable conditions are still harsh. For typical plain concrete and lightweight aggregate concrete, the percentage of various materials varies.

The basic equation for concrete shrinkage is given by

(A1) $$\varepsilon {\left(t\right)}_{0}\;=\;\frac{t}{152.79\;+\;3.27t}\;\times \;10^{-3}$$

The basic equation for concrete creep is given by

(A2) $$\varphi {\left(t\right)}_{0}\;=\;\frac{t^{0.6}}{4.168\;+\;0.312t^{0.6}}$$

(2) ACI Model

ACI 209 (92) has been widely used for calculating concrete deformation accurately. This method first relies on environmental factors to materialize the ultimate creep coefficient of concrete and then fine-tune the creep according to time. In this model, the correction coefficient of the concrete is mainly used, but since the parameters cannot be specified in the design, the correction coefficient is usually not expressed in the design stage. The model can determine the creep of concrete under unconventional conditions.

The basic equation for concrete shrinkage is given by

(A3) $$\varepsilon_{s}\left(t,t_{s}\right)\;=\;\varepsilon_{shu}\frac{t\;-\;t_{s}}{H\;+\;t\;-\;t_{s}}$$

(A4) $$\varepsilon_{shu}\;=\;780\;\times \;10^{-6}\cdot \gamma_{sh}\;=\;780\;\times \;10^{-6}\gamma_{RH}\gamma_{vs}\gamma_{s}\gamma_{\psi }\gamma_{c}\gamma_{\alpha }$$

The basic equation for concrete creep is given by

(A5) $$\varphi \left(t,\tau \right)\;=\;\varphi_{u}\frac{{\left(t\;-\;\tau \right)}^{0.6}}{10\;+\;{\left(t\;-\;\tau \right)}^{0.6}}$$

(A6) $$\varphi_{u}\;=\;2.35\gamma_{cr}\;=\;2.35\gamma_{\tau }\gamma_{RH}\gamma_{vs}\gamma_{s}\gamma_{\psi }\gamma_{\alpha }$$

(3) CEB-FIP (1978) model

The average shrinkage strain of concrete occurring during the time interval is

(A7) $$\varepsilon_{sh}\left(t,t_{0}\right)\;=\;\varepsilon_{sh0}\left[\beta_{sh}\left(t\right)\;-\;\beta_{sh}\left(t_{0}\right)\right],\varepsilon_{sh0}\;=\;\varepsilon_{sh1}\varepsilon_{sh2}$$

where

(A8) $$\varepsilon_{sh1}\;=\;\{\begin{array}{cc} 0.333\left(0.41\lambda^{2}\;-\;37.1\lambda \;-\;372\right)\;\times \;10^{-6} & \lambda \;\leq \;98\\ 100\;\times \;10^{-6} & \lambda \;=\;100 \end{array}$$

(A9) $$\varepsilon_{sh2}\;=\;0.7\;+\;1.42\exp\left(-0.18h_{0}^{0.45}\right)$$

(A10) $$\beta_{sh}\left(t\right)\;=\;\{\begin{array}{cc} \frac{t^{0.8}}{t^{0.8}\;+\;0.25h_{0}} & 50mm\;\leq \;h_{0}\;\leq \;600\\ \frac{t^{1.25}}{t^{1.25}\;+\;8h_{0}} & 600mm\;\leq \;h_{0}\;\leq \;1600 \end{array}$$

For a uniaxially loaded concrete member subjected to the constant stress of size *σ_0_* at time *τ*, the creep strain *ε_c_(t,τ)* expression at time *t* is

(A11) $$\varepsilon_{c}\left(t,\tau \right)\;=\;\frac{\sigma_{0}}{E_{c}\left(28\right)}\phi \left(t,\tau \right)$$

(A12) $$\phi \left(t,\tau \right)\;=\;\phi_{d}\beta_{d}\left(t,\tau \right)\;+\;\beta_{\alpha }\left[\beta_{f}\left(t\right)\;-\;\beta_{f}\left(\tau \right)\right]$$

(4) CEB-FIP (1990) model

The calculation equation of the average shrinkage (or expansion) strain of plain concrete members under unloaded condition is

(A13) $$\varepsilon_{cs}\left(t,t_{s}\right)\;=\;\varepsilon_{cso}\beta_{s}\left(t\;-\;t_{s}\right)$$

(A14) $$\;\varepsilon_{cso}=\beta_{RH}\left[160\;+\;\beta_{SC}\left(90\;-\;f_{c}\right)\right]\;\times \;10^{-6}$$

The coefficient of shrinkage strain as a function of time is

(A15) $$\beta_{s}\left(t\;-\;t_{s}\right)\;=\;\sqrt{\frac{\left(t\;-\;t_{s}\right)}{0.035{\left(\frac{2A_{c}}{u}\right)}^{2}\;+\;\left(t\;-\;t_{s}\right)}}$$

The concrete creep coefficient is

(A16) $$\phi \left(t,t_{0}\right)\;=\;\phi \left(\infty ,t_{0}\right)\beta_{c}\left(t\;-\;t_{0}\right)$$

(5) BP model

This method is used to evaluate the empirical formula of the basic creep and the total creep value. This method is quite similar to the ACI model described above. The specific expression is as follows.

The creep function (including elastic deformation) is given by

(A17) $$\Phi_{b}\left(t,\tau \right)\;=\;\frac{1}{E^{\prime }}\left[1\;+\;\phi_{b}^{\prime }\left(t,\tau \right)\right]$$

(A18) $$\frac{1}{E\prime }\;=\;0.01306\;+\;3.203R_{cy}^{-2}$$

The total creep function Φ*_T_*(*t*,*τ*), including elastic deformation, basic creep and dry creep, is given by

(A19) $$\Phi_{T}\left(t,\tau \right)\;=\;\phi_{b}\left(t,\tau \right)\;+\;\phi_{d}^{\prime }\left(t,\tau ,t_{sh,0}\right)/E^{\prime }$$

(6) GL2000

Concrete shrinkage strain can be calculated by

(A20) $$\varepsilon_{sh}\;=\;\varepsilon_{shu}\beta \left(h\right)\beta \left(t\right)$$

Concrete creep is calculated by

(A21) $$creep\;=\;\frac{\phi_{28}}{E_{cm28}}$$

Symbols used in equations of Appendix A are presented in Table A1.

**Table A1:** Symbols in Equations of Appendix A.

Symbols	Meaning
*t*	Calculating age (days)
*t_s_*	Age of concrete at the beginning of shrinkage (days)
*H*	Coefficients related to curing conditions
*γ_RH_*	Influence coefficient of relative humidity on shrinkage
*γ_vs_*	Influence coefficient of body surface contrast contraction
*γ_s_*	Influence coefficient of slump on shrinkage
*γ_ψ_*	Influence coefficient of sand content on shrinkage
*γ_c_*	Influence coefficient of cement content on shrinkage
*γ_α_*	Effect of air content on shrinkage
*γ_τ_*	Loading age influence coefficient
*τ*	time
*λ*	Relative humidity (%)
*h_0_*	Nominal thickness (mm)
*E_c28_*	28-day elastic modulus of concrete
*ϕ_d_β_d_*(*t*,*τ*)	Recoverable hysteresis elastic deformation
*β_α_*[*β_f_*(*t*) − *β_f_*(*τ*)]	Irreversible rheological deformation
*β_RH_*	Coefficients related to the relative humidity of the environment
*β_sc_*	Coefficients related to cement varieties
*f_c_*	Cylinder compressive strength of concrete (N/mm^2^)
*A_c_*	Component cross-sectional area (mm^2^)
*u*	The circumference of the component section exposed to air (mm)
*ϕ* (*∞*, *t_0_*)	Parameters related to concrete compressive strength and ambient relative humidity
*β_c_*[*t* − *t_0_*]	Coefficient of creep over time
$\phi_{b}^{\prime }$(*t*, *τ*)	Basic creep coefficient (10^−3^/MPa)
*R_cy_*	Compressive strength (MPa) of cylindrical specimens of 28d age
$\phi_{d}^{\prime }$(*t*, *τ*, *t_sh_*_,0_)	Dry creep coefficient
*h*	Relative humidity
*ε_shu_*	Coefficients related to cement type and average 20d cylinder compressive strength
*Φ*_28_	Creep coefficient
*E_cm28_*	28-day modulus of elasticity of concrete (MPa)
